# How is autonomy supported for people with dementia living in a nursing home, to what extent and under what circumstances? A realist evaluation

**DOI:** 10.1186/s12913-025-12349-w

**Published:** 2025-02-12

**Authors:** Henny van der Weide, Marleen H. Lovink, Katrien G. Luijkx, Debby L. Gerritsen

**Affiliations:** 1 Department of Primary and Community Care, Research Institute for Medical Innovation, Radboud University Medical Center, Radboudumc Alzheimer Center, University Knowledge Network for Older Adult Care Nijmegen (UKON), Nijmegen, The Netherlands; 2 Archipel Zorggroep, Eindhoven, The Netherlands; 3 Department of Tranzo, Tilburg School of Social and Behavioural Sciences, Tilburg University, Tilburg, Netherlands

**Keywords:** Autonomy, Dementia, Nursing home, Realist action study, Working routines, Relationships, Residents, Family members, Professionals

## Abstract

**Background:**

Being autonomous is important for people with dementia living in nursing homes. Our recent realist review indicated that supporting their autonomy depends on various aspects.

**Objective:**

This study aimed to uncover how people with dementia, their family members and care and treatment professionals experience the support of autonomy in daily care practice: what works, to what extent and under what circumstances.

**Design:**

A realist evaluation was performed using qualitative methods.

**Methods:**

We applied a realist approach through interviews with family members and care and treatment professionals, as well as on-site observations: due to their cognitive condition we could not exchange mutual views with residents directly. We performed these interviews and observations on site to find out how, to what extent and under what circumstances, supporting autonomy interventions work in daily practice situations. Causal assumptions were derived from the empirical data, leading to Context (C) –Mechanism (M) – Outcome (O) configurations.

**Results:**

Data extraction from 24 interviews and 8 observations resulted in 19 CMO configurations on four themes: A. Autonomy and boundaries: providing maximum autonomy influenced by safety and health restrictions. B. Organization of daily care processes: the influence of attempting to increase efficiency by working routines. C. Team competences and collaboration: the possibilities of care professionals to acquire the relevant competences and an appropriate level of team collaboration. D. Interaction and relationships: the accomplishment of a working relationship between residents, their family and care and treatment professionals.

**Conclusion:**

The results showed that supporting autonomy was valued highly by all stakeholders. In streamlining care processes, working routines were influential to supporting autonomy. Weighing risky choices for people with dementia in their decision making was another factor. Our study indicated that realizing autonomy is facilitated by a capable and collaborative team of professionals and by a working relationship between persons living with dementia, family members and professionals.

**Supplementary Information:**

The online version contains supplementary material available at 10.1186/s12913-025-12349-w.

## Background and objective

Worldwide, 55 million people are affected by dementia, a figure that is predicted to increase to around 80 million in 2030 and to 132 million by the year 2050 (www.alz.org). Dementia is a degenerative disorder that, over time, results in significant cognitive decline and the need for assistance with activities of daily living. As dementia progresses, assistance will further be needed in making decisions, which is connected to the issue of how to remain autonomous despite significant cognitive impairment. Autonomy is a complex notion and several definitions have been formulated. For our study we adopt the definition of van Loon: “Autonomy is a capacity to influence the environment and make decisions irrespective of having executional autonomy, to live the kind of life someone desires to live in the face of diminishing social, physical and/or cognitive resources and dependency, and it develops in relationships” [19].

Throughout the world there is an ongoing public debate on how to maintain autonomy for all individuals: experiencing a true sense of autonomy adds to the quality of life and a feeling of well-being. The European Union e.g. states in a Think Tank briefing of March 21, 2021 that the ongoing debate is wider and entails all human beings in all sectors of society. For people with dementia relying on long term care and living in nursing homes, the right to live your life as you wish becomes even more difficult to maintain [11]. Nevertheless, people with dementia still wish to make their own decisions and they are, each to a different extent, still able to do so [8]. The influence of their health situation, however, complicates the way in which they can express their preferences and wishes.

In a recent rapid realist review we explored what is known about autonomy interventions for people with dementia in nursing homes: what works, in which context, how and why [17]. Causal assumptions were derived from 16 selected articles. The results showed that autonomy can be successfully supported when relevant aspects are considered: the skills of care and treatment professionals, the personal characteristics and competences of residents and those of their family members. Supporting autonomy therefore, seemed to be an interactive process that needs the expertise and support of family members and professionals to be effective. To underpin the theoretical results of the rapid realist review, we used a realist perspective to explore the complexity of daily care experiences. We collected and analysed empirical data in-depth to enhance our knowledge and understanding of daily care situations on the support of autonomy.

Our study thus attempted to uncover how people with dementia, their family members and professionals experience the support of autonomy in daily care practice: what works, for whom, to what extent and under what circumstances?

## Methods

### Realist evaluation

To explore how people with dementia living in a nursing home, their family and care and treatment professionals experience (the support of) their autonomy today, we performed a realist evaluation. A realist approach tries to verify not so much the answer to the question: “does it work?”, but it foremost tries to enlighten how it works, for whom it works, to what degree and under what circumstances. The realist philosophy acknowledges that all data collected are shaped and filtered through the human brain [21]. Therefore this study does not lead to a final truth, but to a better understanding of a complex reality. According to Pawson and Tilley [12], realist studies start with, and are based on, initial hypotheses on how and why an intervention may or may not work, in which contexts, and what mechanism triggers lead to particular outcomes. These hypotheses in its turn, take the shape of a CMO configuration. The realist approach involves the search for causal relations between contexts (C), mechanisms (M), and outcomes (O) [16]. Realism therefore has an explanatory focus and aims to uncover the mechanisms of complex interventions, with particular reference to contexts (see Table 1 for working definitions of key elements). A context-mechanism-outcome configuration is central to analysis and the theory development for realist studies [5].

**Table 1:**
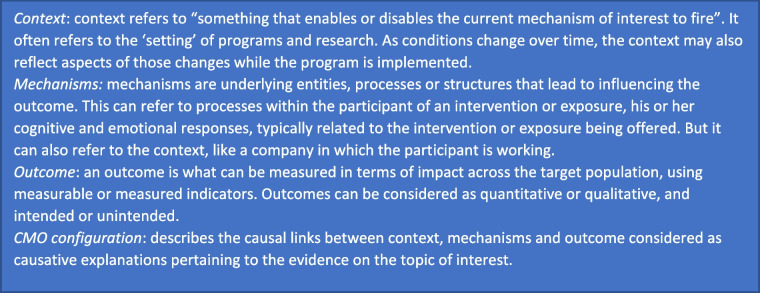
Definition of realist terms [18]

### Setting

Our study was performed among people with dementia living in four small scale units in a nursing facility to provide more insight into how people with dementia living in a nursing home, their family and professionals experience the support of autonomy. The living units were selected to reflect an average nursing home context in the Netherlands: a nursing home in a medium suburban area, providing small scale living arrangements to older people with dementia needing 24/7 assistance and care in sheltered housing. Caring for people with dementia in the Netherlands is preferably provided in smaller scale living environments to prevent over-stimulation and to facilitate more personal attention to residents [3, 20]. Unique for the Netherlands is that specifically trained elderly care physicians provide medical care for these nursing home residents. Both specialists and therapists are commonly employed by the nursing home organization. Most of the round-the-clock direct care in nursing homes is provided by care and treatment professionals, including registered nurses, certified nurse assistants and nurse aids [1]. Our research was performed in four residential units, all belonging to a large organization. Three units were situated on the ground floor of a location consisting of in total 15 small-scale living units. These three units participating in our study were physically connected by a substantial garden space. Each living unit consists of 10–12 bedrooms, with shared bathrooms. A unit has its own (shared) living quarters. Teams worked independently on a unit, but they assisted each other when necessary. In addition to these three units, our research was also performed in a fourth unit at another location, associated with the same organization. This small-scale residential unit is situated in a little village and stands alone within a different care context (intellectual disability care). It provides a family-like environment for seven residents with dementia. The individual bedrooms each have a bathroom.

### Study design and data collection instruments

This study was performed through individual interviews and on-site observations. Per residential unit, three resident-representatives or family members and three care and treatment professionals (registered nurses or certified nurse assistants and/or (para)medics) were randomly chosen and verbally invited to participate. They consented to an interview on their views and experiences on the support of autonomy of the people living in their unit. Two interview guides were developed, one for residents’ representatives and another one for professionals (Appendices 1 and 2).

The interview guides stated questions on both family members’ and professionals’ views on the importance of residents’ autonomy, the way they thought residents experienced it to be and what they thought could be done to improve the support on autonomy.

All guides were discussed, refined and established by the research team. To introduce a perspective on autonomy as a notion, we presented the interviewee with a few examples from our review. By using results closely connected to the everyday world of a nursing home, the interviewees were able to connect to our questions. An interview was performed by one of two researchers (HvdW, IC) and took about an hour each. They were recorded and transcribed verbatim for analysis. In addition to the interviews, two three-hour observations per unit on site were scheduled and conducted by two researchers alternately (HvdW, IC). In observing everyday care activities in a nursing home, we concentrated on the context, the interaction of people involved and the outcome on autonomy (Appendix 3). Interaction between clients was described preserving their anonymity.

The observations were all performed from a back seat in the living quarters of each unit by the researcher who made herself known beforehand. Observations were fully reported and researchers reflected on the results.

### Participants and informed consent

The sample consisted of care and treatment professionals, relatives and people with dementia living in four different dementia special care units of a nursing home in the southern part of the Netherlands. Eligibility to a nursing home in the Netherlands requires residents to have an official declaration of a dementia diagnosis. All residents met this inclusion criterion. Care and treatment professionals of the selected wards were included in the study when working during observations and when interviewed. We did not make any observations during the intimate personal (morning or evening) care, we only observed group care in a shared living quarters. No names of residents of the participating living units were recorded during observations and no actions were imposed on them. All stakeholders, including residents, were invited to the introduction of our study on several occasions. Residents however were not present. We aimed to recruit all residents of the participating living units in the nursing home. Each residential unit has a team of care and care and treatment professionals that are responsible for the daily care of residents. The number of employees ranged to about 15 (care) professionals per residential unit. We aimed to recruit them all to our study. First we invited potential participants of this study to two information meetings. They were carefully informed to be sure that they were able to fully make up their minds about the nature of (participating in) this research. When needed and on request these meetings and this letter of information were followed by (further) clarification by phone or face to face.

We individually asked their legal representatives for informed consent for themselves and for their family member. On account of their cognitive condition, residents were not able to give an informed consent for themselves so, in accordance with Dutch law, their legal representatives were formally asked to do so.

We also asked the care and treatment professionals for their informed consent. After the information meetings we sent an informed consent document to all potential participants to sign and return to the first researcher.

### Data analysis

Interview transcripts and observation reports were analysed similarly and data were directly coded into CMO configurations [9]. To facilitate direct coding into the development of C-M–O configurations, Jackson and Kolla were the first to propose identification of connections of context, mechanisms and outcome elements during the coding process; we used their technique to analyse the links between contexts (C), mechanisms (M) and outcomes (O) directly from the texts.

The definitions of contexts, mechanisms and outcomes as used in our review were adopted in this study to ensure consistency and transparency (see Table 1). Three researchers individually performed the coding process for the first two interviews (HvdW, ML and IC). After discussing the results, the research team agreed on the coding process.

The identifying and analysing process was further performed through abductive reasoning, using realist logic to find the simplest and most likely conclusion [21] by one researcher (HvdW), checked by two other members of our research team (ML, IC).

### Appraisal

The analysing process resulted in 186 causal connections (CMOs) on four living units. After removing doubles *per* living unit and subsequently matching and replenishing CMOs *per* living unit, we were able to reduce the number of CMOs on four units to 91.

The next analysing phase involved prioritizing the configurations giving a highlight to the focus of our study: supporting autonomy in everyday care situations. Two reviewers (HvdW; ML) established the causal connections that had the most relevant focus and could therefore contribute to our theory development; considerations of relevance and rigour were applied fitting the aim of this study.

For relevance and rigour we used two specific questions to decide whether to include the data: do the data help to refine or substantiate our research question? And: are the data of added value to our research question? (www.ramesesproject.org, 2014).

To be able to perform this process consistently, the research team (HvdW; ML; DG; KL) defined specific criteria for the exclusion of CMOs for reasons of specific themes, such as sexuality, freedom of mobility, end of life decisions. These themes are closely connected to the concept of autonomy, but the main focus of these studies was specifically on the indicated themes, not on autonomy.

For this study we excluded CMOs with a focus on:

- Definition of autonomy;
- Physical living conditions;
- Issues of Life and death;
- Sexuality;
- Freedom of mobility;
- Transfer into a nursing home.

The excluding decisions were made in a meeting with three researchers (DG, ML, HvdW). Its results were approved in the full research team (HvdW, ML, IC, DG, KL) and led to the exclusion of 18 CMOs, leaving 73 CMOs on all four residential units together.

After that we removed doubles over all four living units leaving a remaining total of 39 CMOs.

**Fig. 1:**
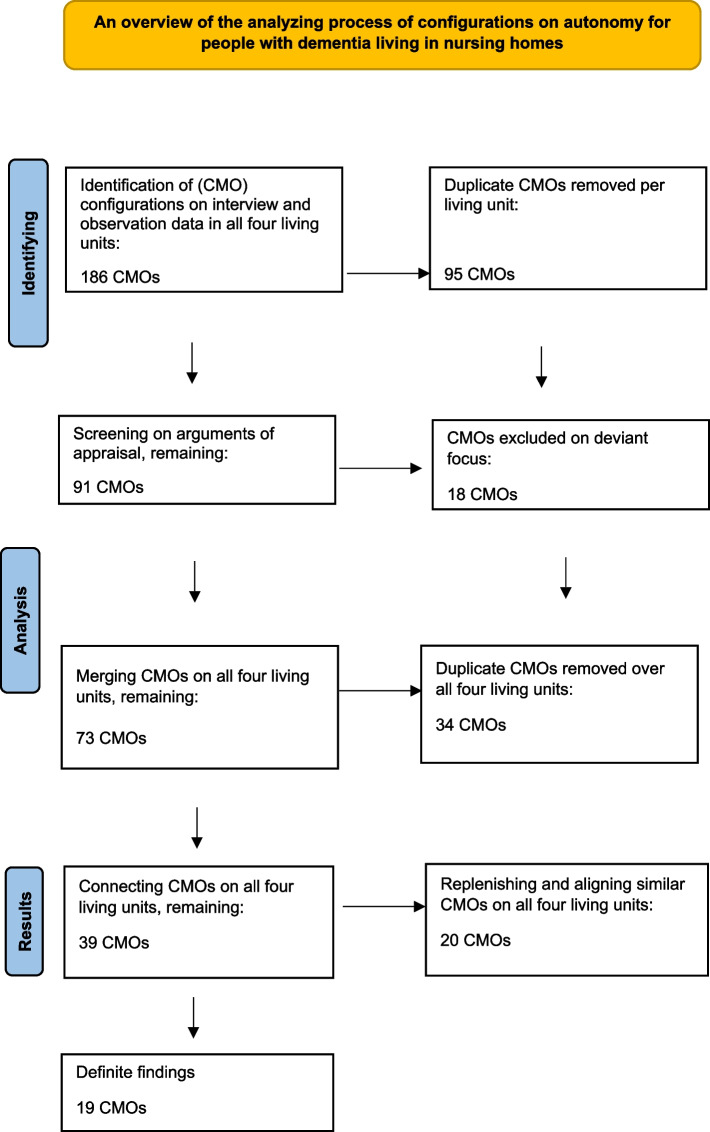
Analysis flowchart

By arranging the results, similar and complementary CMO configurations emerged. We replenished and aligned these CMOs on all four living units together having the same focus, preserving different perspectives. This ultimately resulted in 19 remaining CMOs over four living units (see Fig. 1). On a more abstract level we classified CMO coherence and linked the remaining 19 CMO configurations by grouping the CMO configurations addressing the same topic into four themes.

The results of this phase of analysis were established by three members of our research team (HvdW, ML, DG) and were approved by our research team as a whole.

## Results

### Context

Our study was performed in four residential units of a suburban nursing home. Unit one, two and three are similarly designed and provide a home for a group of 10 to 12 people with dementia. In a building containing 15 residential units, a sense and influence of an organization was noticeable through the presence of a considerable number of other care teams. Residential unit four was situated in a nearby village, in a stand-alone context. It provides a more family-like living setting for seven people with dementia. Family members said to be closely connected to the village, sometimes co-working with the team. This care team introduced themselves as a quite independent team of professionals since there were no other teams around to support them. The contexts of all units were similar as regards team composition and target group: people with dementia.

### Participants

Sixty-six professionals were asked to participate and consented to their participation. Forty-nine residents’ representatives were asked for an informed consent; forty-four family members consented and five residents’ representatives did not consent and refused participation for themselves and for their family member. No data were recorded on these residents and relatives. The number of participants fluctuated somewhat during the performance of our study due to an overturn on all residential units of residents and of care and treatment professionals. Age range in residents varied from 76 to 95 years old, one resident had a foreign cultural background. The team of professionals also varied during the process of our study in gender, in age and in background. Nine professionals had a foreign cultural background. On an average, care professionals had nursing experience (with years ranging from 1 to 24).

We performed 24 interviews, six interviews per living unit: three health professionals and three family members. Overall, twenty-one participants were females, three participants were male. Professionals had mixed positions: two contact-nurses (being the contact person for residents and their family members), one nurse, two certified nurse assistants, one physiotherapist, one team coach, one medical doctor, one nurse specialist and four residential care assistants. Family members were variously related to a resident: five participants were wives, three were daughters, a sister, a niece and a son-in-law. During our eight observations – two observations per residential unit—eight to twelve clients were present per living quarters, their presence varying during the day because of daily activities. Four care professionals were generally working: one certified nurse assistant or certified nurse, two nurse assistants and one residential care assistant per residential unit.

### Themes

After analysing all data, four themes were identified:

- Maximum autonomy and some boundaries
  The intent to realize as much autonomy for residents as possible, balanced by considerations of wellbeing;
- Organization of daily care processes
  The influence of an attempt to increase the efficiency of providing everyday care;
- Team competences and collaboration
  The awareness of care professionals to acquire the relevant competences and reach an appropriate level of team collaboration;
- Interaction and relationships
  The accomplishment of a working relationship between residents, their family and care and treatment professionals.

Below we present how each theme is explained by CMO configurations showing different aspects of this theme. The order in which themes were stated does not reflect their importance. Each theme was elaborated on by adding detailed results to the identified CMOs, where further explanation was considered helpful for a better understanding. Some of the CMOs were illustrated by a relevant quote We chose not to state specific residential units for reasons of traceability. Occasionally CMOs were found from both a positive and negative perspective, depending on the result of a specific residential unit. In the table this was indicated by a broken line (Tables 2, 3, 4, and 5).

**Table 2:**
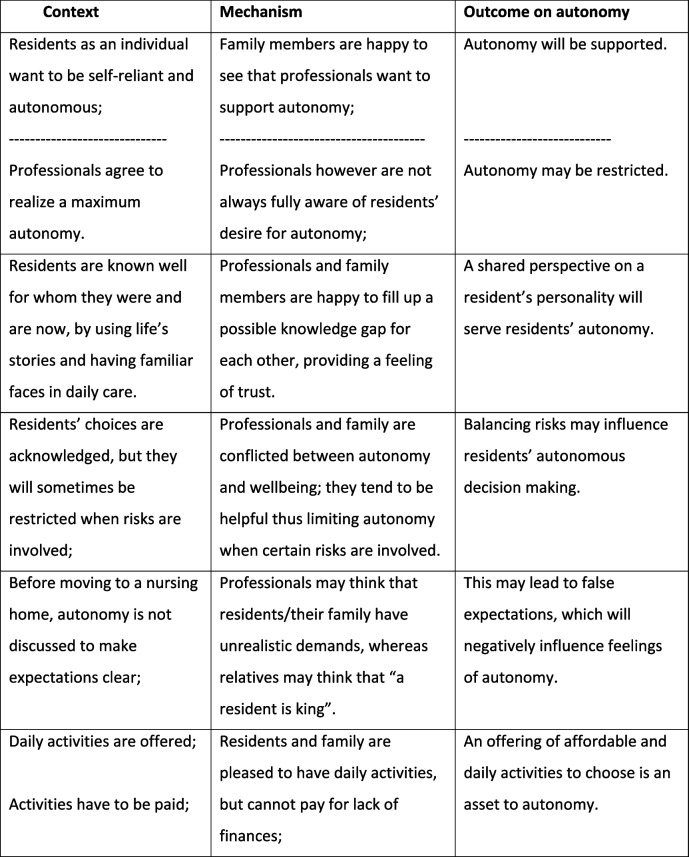
Maximum autonomy and some boundaries

**Table 3:**
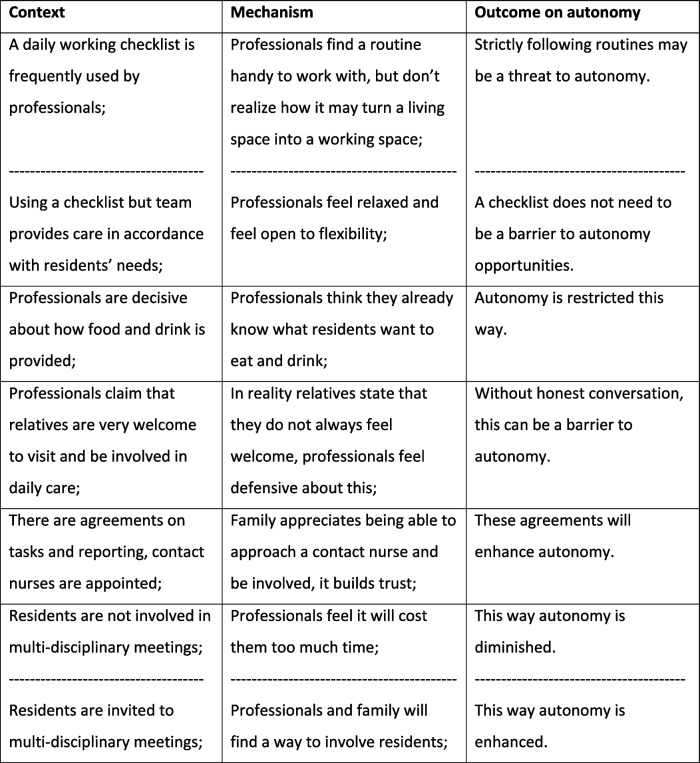
Organization of daily care processes

**Table 4:**
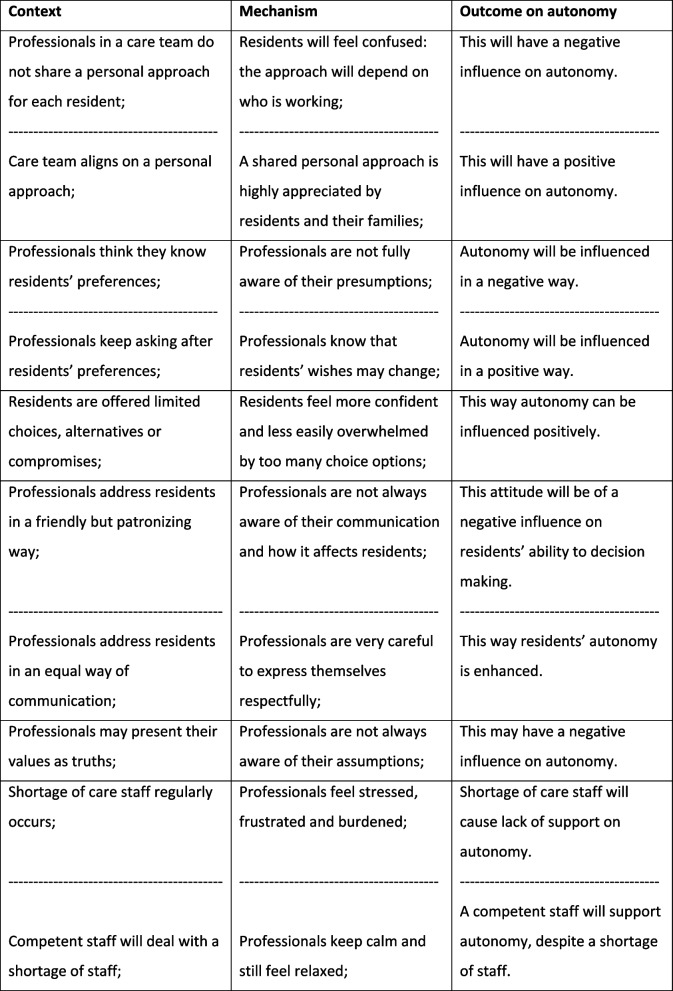
Team competences and collaboration

**Table 5:**
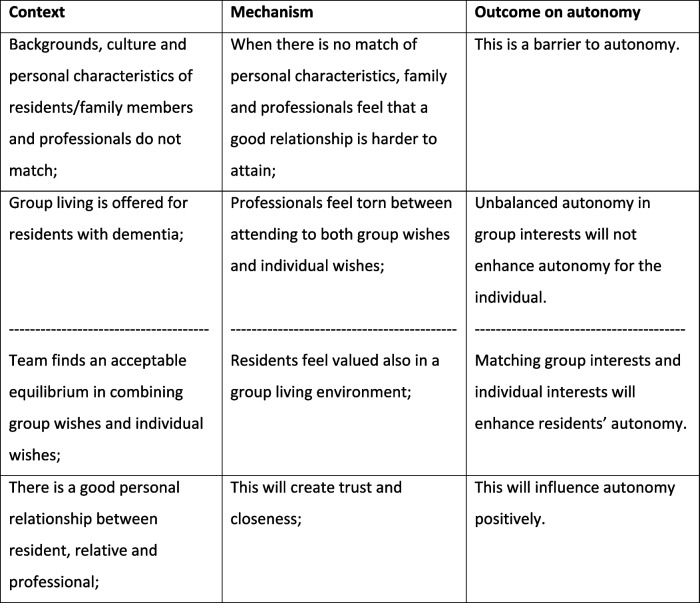
Interaction and relationships

#### Maximum autonomy and some boundaries

We observed a profound wish of people with dementia to feel self-reliant and to be able to live their lives as they did before. A variety of preferences emerged from interviews and observations stating specific personal wishes such as holding on to your own key to the apartment or flexibility in a daily choice of activities.

Preferences and wishes became apparent when residents, family members and professionals exchanged views on who this resident really was as a person, combining residents’ communication and behaviour, adding knowledge on someone from the past and knowledge on seeing someone on a daily basis, thus building a complete picture. Even when knowing a resident really well, professionals would keep asking after preferences, trying to avoid presumptions. Sometimes, frequently inquiring after someone’s preferences, however well-intended, also proved to be tiring for residents.

“However, someone else making an educated guess in deciding for our mother, brings her piece of mind” (030286 – relative)

The impact of autonomy and its daily performance were usually not discussed before moving into the nursing home. For instance, family easily accepted that people with dementia are cared for in a group-living arrangement; but in the course of time group interests proved to be different from individual preferences, which sometimes caused dilemmas in daily care.

“It is a good place to be! But later on we discovered that living in a group also proved to have a downside for our father” (050250 - relative)

Another finding was that there could be risks involved in residents’ decision making. Both relatives and professionals indicated to be conflicted about the acceptance of these risks when decisions involved residents’ safety or when it could be damaging to their wellbeing.

“The lady likes to eat, she tells me. Then who am I to tell her she is not allowed to?” (020436 - professional)

Professionals and relatives stated that balancing risky choices can be of direct influence on the experience of autonomy of residents when others interfere in their decision making. They observed that residents found this difficult to accept, which had an influence on their feeling of well-being.

#### Organization of daily care processes

All care teams used working routines in care processes. Professionals found it useful to commit each other to maintaining a checklist of tasks. It allowed them to feel satisfied when accomplishing their tasks and not missing important actions. But these routines also caused a living unit to become too much of a working place.

It had an impact on flexibility, causing flexibility to become more of a “service” instead of a normality. Routines on daily care, eating and drinking procedures and team arrangements on professional tasks sometimes led to losing sight of residents’ individual wishes or needs.

Tasks needed to be finished and self-inflicted rules took precedence where residents’ preferences could have prevailed. We observed for example, that delaying lunchtime for half an hour because of residents’ activities was not easy to match with the care and treatment professionals’ lunch break, causing a certain reluctance in the care team to enhance autonomy in this respect.

“Professionals seem to determine the course of events: every day has its fixed format” (010290 - relative)

“There are arrangements on when to change clothes; now there’s something we should not be so strict about” (030474- professional)

Furthermore, routines and inadequate workforce capacity conditions resulted in multi-disciplinary meetings regularly being held in absence of residents. Although organization policy stated that residents must be offered the possibility of attending these meetings, routines tended to avoid their presence, usually based on the state of a resident’s dementia and due to the care professionals’ (presumed) lack of time. Relatives were usually invited to represent their family member. Our findings showed a mixed picture on this aspect, where some care teams made more effort in having a resident present than others.

“To be honest, residents are not invited to multi-disciplinary meetings while some would probably be able to tell us something about their preferences we are not aware of” (030409 - professional)

#### Team competences and collaboration

Team competences were considered to be an important factor in providing autonomy for residents with dementia in a nursing home. Adequate staffing in number and expertise were a decisive factor when providing a personal line of contact for each resident. Professionals showed expertise in disclosing residents’ preferences and wishes despite the presence of dementia by limiting and visualizing choices and finding alternatives. They generally felt competent and responsible and they were motivated to make a success of their team work. However, care professionals were not always aware of their own presumptions and values: they projected their own beliefs out to residents and this way they had an influence on residents’ choices.

“You need to let go of your usual thinking patterns; there is a lesson to learn!” (010417 - professional)

In certain teams, care professionals showed an open and transparent communication, whereas other observations “elderspeak” was illustrated, which is friendly but also patronizing.

Resident seems restless: ”Now please sit down properly” (observation)

This form of communication between professionals and the people with dementia appeared to be influenced by a care professional’s self-reflection competence and that of the care team: we observed a certain non-awareness to this kind of phraseology.

Finally, team members indicated that they thought that team collaboration is usually open minded and constructive.

“ In our team we all share the same goals, motivation and drive. Very important!” (050463 – professional).

#### Interaction and relationships

The fourth theme regarded the interaction and the relationship between resident, family member and professional. A personal connection in mutual relations was found to be an asset of value in creating possibilities for supporting autonomy.

Connecting personalities strengthened trust and had a positive impact on residents’ confidence. Religious beliefs were sometimes found to be a barrier between resident and family for example in case of eating and drinking.

“Allah does not want her to eat pork, but dementia prevents her from remembering, so she will put it in her mouth and this displeases her family” (020413 - professional)

Since providing a Muslima with certain food is unacceptable to their beliefs, relationships would become strained and family would feel unheard in this respect when not constructively discussed. Yet, even when relationships were positive and close, professionals found it difficult to compromise between residents’ wishes, family values and professionals’ possibilities: that way residents’ autonomy was influenced by values other than their own. As mentioned before, group-living sometimes caused a strain on relationships: group interests were easily conflicted when trying to accomplish individual autonomy or vice versa. There were friendly connections between residents living in a group setting, but animosity and differences would sometimes be enlarged, which could lead to individual agitation.

## Discussion

In this study we aimed to uncover how people with dementia, their family members and professionals experience the actual (support of) autonomy in daily care practice. Context-mechanism-outcome configurations were identified pertaining to four themes: Maximum autonomy and some boundaries; Organization of daily processes; Team competences and collaboration and Interaction and relationships. Our interviews show that residents, relatives and professionals in care teams value the autonomy of residents: in interviews relatives and professionals indicated that residents generally still wished to make their own decisions. Residents, relatives and professionals in care teams value the autonomy of residents: residents generally wished to make their own decisions as much as possible, relatives felt the need to be closely involved in decision making and professionals usually considered it their mission to support residents and help them feel as self-reliant and independent as possible.

We found, however, that in daily care, operational structures and processes could stand in the way of a residents’ free choice. Although daily routines could improve care processes, they also showed to be a barrier to support autonomy when professionals tried to make their work increasingly efficient. In line with Custers et al. [2]’ study, we also found that residents and their relatives frequently accepted daily care conditions as they were and so gradually professionals grew to prevail the daily course of events [2].

It does not serve the support of autonomy, when the focus first and foremost lies on task completion and documentation. Because of these factors, the rhythm of running a nursing home living unit and the practices that might become normalized, could create an environment where residents find that their opportunities to exercise autonomy remain unseen [14]. In our study, professionals, when asked, were well able to reflect on this process, stating it to be a certain level of complacency: they felt like they gradually became used to this feeling of control, though usually they were unaware of this consequence. Our study further showed that team collaboration and competences made a difference when professionals wished to spend time supporting residents on autonomy, both in daily living and also for instance, in involving residents in care meetings. In nursing care today it is a challenge to be adequately staffed and availability of time is not self-evident [10]. It is hard to create space for professionalization and improving collaboration in care for people with dementia, nevertheless this is of great importance for supporting autonomy of people with dementia. Timesaving strategies, (not) having adequate time to reflect and learn and providing residents with the opportunity to make decisions at one’s own pace has been highlighted earlier [6].

This issue may need further attention in how to maintain adequate standards of providing autonomy to people with dementia living in nursing homes in the Netherlands.

In our rapid realist review [17] we analysed results of published studies involving the support of autonomy for people with dementia living in nursing homes. Our current study was performed to investigate this issue empirically viewed from a realist perspective, specifically because few of the reviewed publications had used this perspective. The themes which emerged from our current study showed similarities to those of our review (being: Preference and choice; Personal characteristics of residents and family; Competent nursing staff and Interaction and relationships). Matching personalities and close personal relationships, adequate expertise and staffing conditions are issues we found in both studies. For example, as in our review, we found that a match in character of the persons involved makes a difference and positive relationships have an impact on how autonomy can be supported and realized.

A positive relationship between residents-relatives and professionals based on respect and trust, turned out to be an important factor. Frequently changing relations will be complicating: caring is a dynamic process and it demands knowing each other to enable continuous involvement and decision making [15]. In reality, the overturn in both residents and professionals is increasing. This development may very well put a considerable strain on the support of autonomy in dementia care.

But differences between the results of our review and our present study were also found. Using the realist approach, our current study showed that some of our recent findings seemed to have a higher impact in daily care situations than was found in our review. We noticed for instance that a residents’ decision making was found to be limited on a day-to-day basis when professionals thought risks were involved. In our review we excluded certain situations of limiting autonomy. In our current study, we could not easily dismiss limitations to a residents’ risky decision when observing everyday care situations. More often than expected, consequences of dementia in daily life causes dilemmas in how to balance autonomy with a residents’ safety and wellbeing [11]. Residents are usually able to indicate their preferences to a certain extent, but their condition regularly urged professionals and relatives to reconsider these wishes and weigh the risks involved. Both parties struggled with these considerations and tried to find risk-free alternatives that could also satisfy the needs of residents. This kind of taking over a residents’ decision making – to a certain extent – was indicated to be usually based on the grounds of just being helpful, drawing a less rigid distinction between autonomy considerations and best interest judgements.

Moreover, these considerations sometimes also resulted in overlooking the possibility of including residents with dementia in important decision making during multi-disciplinary meetings. This result could be unfortunate, for important insights into residents’ preferences can commonly still be revealed when “listening” actively to their expressions [13]. A resident’s social wellbeing on the other hand, could also ask for a different approach. Possibly we may sometimes overestimate autonomy wishes where people with dementia are concerned [4]. Interviewees indicated that some residents felt more comfortable by not having to make decisions all the time. If so, this feeling of comfort seemed to be valued more than the support on autonomy by continuously inquiring after individual preferences.

In addition, our recent study demonstrated that in general family members were acceptant of protective measures for a resident and professionals usually accepted this accordance [11], both striving to remain on the same side [7]. Contrary to the findings in our review, we did not find an explicit desire of relatives to become more equipped in dementia expertise in multi-disciplinary meetings or other encounters. It did not seem to play a significant role and family members did not specifically express a feeling of individual inequality in relations: they stated that they trust care and treatment professionals to level the gap in knowledge by offering more information if necessary. This feeling could also be rooted in a desire to maintain friendly connections. More research in this field could enlighten our findings.

Comparing our studies we may conclude that a realist perspective enlightens the added value of exploring the support of autonomy for people with dementia living in nursing homes.

### Strengths and limitations

First of all, our study was to a certain extent limited because we could not explore the resident’s desire for autonomy directly in an interview, due to their cognitive condition. We did however try to ensure our findings to be as valid as possible by interviewing their representative family member, the professionals and our observations. Our study was also limited by the fact that it was performed within one organization in the Netherlands. A large organization indeed, but still the contexts were more or less alike for all four living units and offered no great variety in circumstances, except for the stand-alone situation of one of the units. We might, for instance, have found another degree of influence of working routines in other organizations. Yet this study offered an opportunity to collect in-depth data on supporting autonomy and we found different results in rather similar contexts. We explored layers in context: below the surface people’s characteristics and relationships differ in every situation, which make context factors highly variable. Another limitation is that we have probably influenced our results by excluding decision making with another focus than daily care situations. Focussing on daily care however, can also be regarded a strength because the core of supporting autonomy primarily lies in those daily care situations. Using a realist evaluation approach strengthened our study, helping us to better understand a complex practice situation, leading to an improved theory development.

## Conclusion

We identified 19 CMO configurations on four different themes, showing that supporting autonomy for people with dementia in nursing homes was observed to be valued by residents as was confirmed by indications of relatives and care and treatment professionals. We found that working routines can have a considerable impact on the support of autonomy for people with dementia. Weighing risky choices by professionals and relatives also proved to be of influence when residents wish to remain autonomous, limiting a resident’s decision making when they considered this decision hazardous. Our study indicates that realizing autonomy is strongly facilitated by the way in which a team is capable and collaborative and by a positive relationship, where resident, family and professional all have a fair perception of each other as a person, understand different points of view, thus being able to build a relationship of trust.

## Supplementary Information

Supplementary Material 1.

Supplementary Material 2.

Supplementary Material 3.

## Data Availability

Data is provided within the manuscript or supplementary information and on reasonable request at the corresponding author.
