# Supplementary material for: How is autonomy supported for people with dementia living in a nursing home, to what extent and under what circumstances? A realist evaluation
Source: BMC Health Serv Res. 2025 Feb 12;25:237. doi: 10.1186/s12913-025-12349-w (PMC11817305; doi:10.1186/s12913-025-12349-w)
Supplement: Supplementary file 1 — Supplementary Material 1. [file 12913_2025_12349_MOESM1_ESM.docx]

**Appendix 1**

**Semi-structured INTERVIEW GUIDE** (*relatives*)

“How is autonomy supported for people with dementia living in a nursing home, to what extent and under what circumstances? A realist evaluation”

1. Could you please tell me about your involvement in this nursing home and on this residential unit? What is your opinion and what are your feelings about being here? Are other family members also involved and how?
2. How does your relative perceive his or her life in this nursing home?
3. This interview is about supporting autonomy for people with dementia living in a nursing home. How would you describe autonomy and what does it mean to you? In what way do you perceive the daily performance of autonomy in this unit?
4. In literature maintaining autonomy is considered a personal feeling. Do you agree? Could you tell us which aspect of autonomy you think your relative would consider most important?
5. What is most important to address in supporting autonomy for your relative in this residential unit? When would you be content?
6. I would like to inspire you by presenting a few results from our literature review. Do you have any additional examples? What aspect, to your opinion, is important to pay attention to in this study?

Example 1:

“Autonomy will be considerably improved through the presence of clients at multi-disciplinary meetings. These meeting should also include daily care decision-making.”

Example 2:

“Besides knowing clients really well, professionals should also know the importance of asking further questions about preferences and wishes to avoid misunderstandings or changes of opinion.”

Example 3:

“Flexibility and creativity are competences you need to find possibilities to realize clients’ preferences.”

Example 4:

“Supporting autonomy may lead to dilemmas due to different interests of clients, relatives and professionals.”
