# Supplementary material for: How is autonomy supported for people with dementia living in a nursing home, to what extent and under what circumstances? A realist evaluation
Source: BMC Health Serv Res. 2025 Feb 12;25:237. doi: 10.1186/s12913-025-12349-w (PMC11817305; doi:10.1186/s12913-025-12349-w)
Supplement: Supplementary file 2 — Supplementary Material 2. [file 12913_2025_12349_MOESM2_ESM.docx]

**Appendix 2**

**Semi-structured INTERVIEW GUIDE** (*care and treatment professionals*)

“How is autonomy supported for people with dementia living in a nursing home, to what extent and under what circumstances? A realist evaluation”

1. How would you define autonomy and what does it mean to you?
2. To your opinion, in what way is autonomy supported on this residential unit and how do you differentiate between individual clients?
3. What is your opinion on the support of autonomy in daily care today? Can you illustrate your answer?
4. How do you yourself support autonomy in practice situations? Can you provide some examples?
5. How do you and your colleagues coordinate clients’ preferences with your own responsibilities; and if that turns out to be difficult, how do you maintain a balance?
6. According to you, what is the most important aspect on supporting autonomy to address in this residential unit? What do you need to attain this goal and could you mention some facilitators and/or barriers?
7. I would like to present a few results from our literature review. What is your opinion on these results? What example appeals to you most? Do you maybe encounter these situations in your work and in what way?

Example 1:

“Autonomy will be considerably improved through the presence of clients at multi-disciplinary meetings. These meeting should also include daily care decision-making.”

Example 2:

“Besides knowing clients really well, professionals should also know the importance of asking further questions about preferences and wishes to avoid misunderstandings or changes of opinion.”

Example 3:

“Flexibility and creativity are competences you need to find possibilities to realise clients’ preferences.”

Example 4:

“Supporting autonomy may lead to dilemmas due to different interests of clients,
