# Supplementary material for: How is autonomy supported for people with dementia living in a nursing home, to what extent and under what circumstances? A realist evaluation
Source: BMC Health Serv Res. 2025 Feb 12;25:237. doi: 10.1186/s12913-025-12349-w (PMC11817305; doi:10.1186/s12913-025-12349-w)
Supplement: Supplementary file 3 — Supplementary Material 3. [file 12913_2025_12349_MOESM3_ESM.docx]

**Appendix 3**

Semi structured observation guide – topic list 2023/2024

Study: It is still my life! Supporting autonomy for people with dementia

Living in a nursing home

Organization and living units:

Frequency of observations: Two observations per unit

Times of observations: Per unit during varying times between 10 pm until 18 pm

Duration of observations: Three to four hours per observation

Place of observations: Shared living space of the residential unit

Researcher: Henny van der Weide, Marleen Lovink, Imke Casimiri (alternately)

Role: Observer (made herself known)

Setting: Residential unit for people with dementia

Contextual information: Description of physical space

The observation data are extensive described and processed in a defined lay-out, matching this checklist. We decided to specifically observe all actions affecting autonomy support. Moreover we will also make notes on general observations relevant to our study theme.

*Focus of observations:*

- Activities, actions and proceedings by residents, staff members and family members present.
- Ordinary activities, actions and proceedings during the day relevant to the support of autonomy of residents with dementia.
- Observation of facilitators and barriers on the support of autonomy for people with dementia
- Observation on the process of communication verbal and non-verbal communication.
- Specific actions performed to support autonomy.

*General observations relating to autonomy:*

1. Participants: what people am I observing?
2. Activities: what actions am I observing?
3. Physical surroundings: is there a relevant physical context that I am observing?
4. Events: what relevant events am I observing?
5. Time schedule: what is the sequence in time and how is this relevant to our study?
6. Purpose and result: what seems to be the purpose of the action that I am observing and what can I really see en tell about the results?
7. Feelings: what emotions and feelings am I observing?
8. Me: how does this observation affect me?
